# Extracellular Vesicle-Associated Epithelial–Mesenchymal Plasticity (EMP) as a Driver of Metastatic Evolution and Therapeutic Resistance in Ovarian Cancer

**DOI:** 10.3390/cancers18213595

**Published:** 2026-11-09

**Authors:** Julia N. Hibbard, Ata Ullah, Farzana Alam

**Affiliations:** 1 Department of Pharmacology and Toxicology, University of Utah, Salt Lake City, UT 84112, USA; 2 Department of Biomedical Engineering, University of Utah, Salt Lake City, UT 84112, USA

**Keywords:** ovarian cancer, extracellular vesicles, epithelial–mesenchymal plasticity, epithelial–mesenchymal transition, malignant ascites, peritoneal metastasis, chemoresistance

## Abstract

Ovarian cancer remains a lethal gynecologic malignancy, with high-grade serous ovarian carcinoma (HGSOC) accounting for a large proportion of advanced-stage disease. HGSOC commonly disseminates across the peritoneal cavity, where malignant ascites contain tumour cells, stromal and immune cells, soluble mediators, and extracellular vesicles (EVs). Although epithelial–mesenchymal transition (EMT) has often been used to explain invasive behaviour, tumour cells may occupy reversible hybrid epithelial/mesenchymal states rather than completing a uniform transition. This review synthesizes evidence linking EV-associated communication with epithelial–mesenchymal plasticity (EMP), peritoneal dissemination, and treatment resistance, with particular attention to the strength and context of the evidence. We distinguish endosome-derived exosomes from small EV (sEV) preparations whose biogenesis has not been established, and separate observational associations from functional experiments, animal-model findings, and clinical evidence. Our central contribution is an evidence-calibrated framework connecting EV biology, ascites-associated selection pressures, and dynamic cell states while identifying where causal mechanisms and clinical utility remain unproven. EV-associated cargo and EV-targeting approaches are promising research directions, but neither biomarker performance nor therapeutic benefit should be considered clinically established without independent validation.

## Simple Summary

Ovarian cancer remains one of the deadliest cancers affecting women, largely because it is often diagnosed after it has already spread throughout the abdomen and become resistant to treatment. Researchers have traditionally explained this spread through a process called epithelial–mesenchymal transition, in which cancer cells become more mobile and invasive. However, growing evidence suggests that ovarian cancer cells more commonly exist in flexible intermediate states, known as epithelial–mesenchymal plasticity, that allow them to adapt to changing conditions and survive therapy. This review highlights the role of extracellular vesicles (EVs), a broad class of membrane-bound particles released by cells, including a subset known as exosomes, in promoting these adaptive states. EVs can carry proteins and genetic material between cells, influencing communication within the tumor environment. We discuss how EV-associated signaling may contribute to metastasis and treatment resistance and how EV-associated molecules may serve as future biomarkers and therapeutic targets. Understanding these processes could lead to improved strategies for detecting and treating ovarian cancer.

## 1. Introduction

### 1.1. Ovarian Cancer Mortality and Metastatic Disease

Ovarian cancer causes more deaths than any other gynecologic malignancy, largely because it is usually detected at an advanced stage [1]. Among histologic subtypes, HGSOC predominates, accounting for the majority of cases and deaths and anchoring most mechanistic research into the disease [2]. Survival has improved only marginally over recent decades relative to other solid tumors, underscoring persistent gaps in understanding how the disease disseminates and evades treatment [2].

A defining feature of HGSOC is its metastatic route: rather than relying on hematogenous or lymphatic spread, tumor cells detach from the primary lesion—most often the fallopian tube or ovarian surface—as single cells or spheroids shed directly into the peritoneal cavity, where fluid currents transport them to seed the omentum, peritoneal surfaces, and diaphragm [3]. This transcoelomic dissemination occurs well before clinical detection, explaining why most patients present with disease already spread throughout the abdomen at diagnosis; colonization requires that detached cells survive anchorage-independent suspension, adhere to and disrupt the mesothelial lining, and invade the submesothelial stroma [3].

Central to this process is malignant ascites, a protein- and cell-rich peritoneal fluid that develops in most patients with advanced disease and correlates with poor prognosis [4]. Ascites is not a passive byproduct but an active compartment arising from vascular permeability, lymphatic obstruction, and VEGF-driven fluid leakage; it functions as a suspension medium keeping exfoliated tumor cells, spheroids, mesothelial cells, and immune cells in continuous biochemical contact, while carrying extracellular vesicles and soluble factors that modulate chemoresistance and invasiveness [4,5].

### 1.2. EMT Revisited

**Figure 1:**
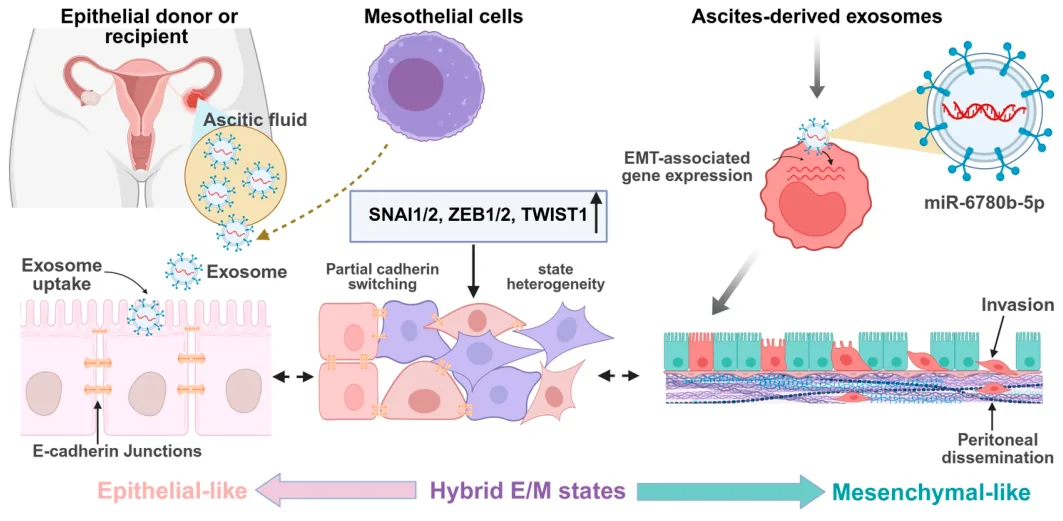
EV-associated induction of EMT/EMP. Schematic depiction of how tumor-derived and ascites-resident EVs drive recipient ovarian cancer cells along the epithelial–mesenchymal plasticity continuum rather than through a single, terminal EMT switch. The left panel shows an epithelial donor/recipient cell retaining E-cadherin-based junctions; upon uptake of EV-associated cargo (microRNAs, proteins, and signaling lipids) released by tumor and mesothelial cells into the ascitic fluid, recipient cells undergo partial cadherin switching and transcription factor induction (SNAI1/2, ZEB1/2, TWIST1), populating a spectrum of hybrid E/M states rather than a discrete mesenchymal endpoint [6,7,8]. The central panel depicts representative hybrid E/M cells co-expressing epithelial and mesenchymal markers, consistent with single-cell transcriptomic evidence of intratumoral state heterogeneity in HGSOC and with the stable, tumorigenic hybrid phenotypes generated experimentally in ovarian cancer models [11,12,13]. The right panel illustrates how ascites-derived EV preparations carrying microRNA cargo such as miR-6780b-5p promote EMT-associated gene expression changes and enhanced invasive/metastatic capacity in recipient cells, closing the loop between the EV-rich ascites microenvironment and plasticity-driven peritoneal dissemination [15,16]. Arrows indicate the reversibility of these transitions, emphasizing that EV exposure shifts cell-state equilibria along the plasticity continuum rather than enforcing an irreversible mesenchymal fate. Created in BioRender. Ata Ullah. (2026) https://bit.ly/4sX48mH (accessed on 6 November 2026).

Historically, the acquisition of migratory and invasive behavior by ovarian carcinoma cells has been attributed to epithelial–mesenchymal transition (EMT), a developmental program co-opted by cancer cells in which epithelial cells lose apicobasal polarity and cell–cell junctions and acquire a spindle-shaped, motile mesenchymal morphology [6]. This transition is orchestrated by core EMT transcription factors, SNAI1 (Snail), SNAI2 (Slug), ZEB1, ZEB2, and TWIST1, acting partly redundantly to repress epithelial genes and activate mesenchymal ones; TWIST1, for instance, requires SNAI2 as an essential downstream mediator to execute EMT and promote metastasis, illustrating this network’s hierarchical nature (Figure 1) [7]. These factors converge on the “cadherin switch,” in which repression of E-cadherin is coupled to induction of N-cadherin, weakening epithelial adhesion while promoting a motile, invasive profile across carcinoma types, including ovarian cancer (Figure 1) [6,8].

Although this binary model, in which a cell is classified as either fully epithelial or fully mesenchymal, has provided a durable framework for carcinoma invasion, it is insufficient to explain key aspects of ovarian cancer metastasis. Ovarian carcinoma cells disseminate as cohesive spheroids that retain epithelial adhesion molecules while exhibiting anchorage-independent survival and invasive capacity, poorly accommodated by a strict epithelial-versus-mesenchymal dichotomy [3]. Complete mesenchymal conversion is rarely observed in patient specimens or ascitic tumor cells, and forcing cells through full EMT can compromise the collective migration and re-epithelialization needed for peritoneal colonization [9]. The linear, terminal EMT model thus captures only the extremes of more continuous, reversible behavior, motivating a broader conceptual framework. The conceptual relationship between EV-associated signaling and reversible epithelial–mesenchymal states is illustrated in Figure 1.

### 1.3. Emergence of EMP

The limitations of the binary EMT model have given rise to epithelial–mesenchymal plasticity (EMP), which posits that carcinoma cells traverse a continuum of hybrid epithelial/mesenchymal (E/M) states rather than switching between two discrete endpoints [10]. Cells in these hybrid states co-express epithelial markers (E-cadherin) alongside mesenchymal markers (vimentin, N-cadherin), retain adhesion sufficient for collective migration, and display plasticity that facilitates invasion followed by re-establishment of epithelial traits at a distant site [9]. These states are not merely transient: engineered hybrid ovarian cancer cell populations retain tumorigenic potential and phenotypic stability over multiple passages, indicating that hybrid E/M identity can be a bona fide, selectable state [11].

Single-cell transcriptomic profiling provides direct evidence for this plasticity in situ. Single-cell RNA sequencing of HGSOC reveals substantial intratumoral heterogeneity, with subpopulations occupying distinct transcriptional states along an epithelial-to-mesenchymal axis rather than two discrete clusters, and mesenchymal- or stromal-enriched subtypes are linked to differences in survival and molecular subtype classification [12,13]. Analogous hybrid phenotypes have been detected in circulating tumor cells co-expressing epithelial and mesenchymal markers outside the ovarian setting, reinforcing that hybrid E/M states are clinically detectable rather than an in vitro curiosity [14]. This evidence repositions EMT from a terminal, cell-intrinsic fate to one node within a broader, tunable plasticity program.

### 1.4. Why Extracellular Vesicles Matter

If EMP is shaped by reversible cell states, extracellular signals may influence transitions between those states. Exosomes are a subset of EVs associated with the endosomal pathway; many studies, however, isolate particles by size or precipitation and do not establish endosomal origin. Such preparations are more conservatively described as small extracellular vesicles (sEVs) or EV-enriched fractions. EV cargo can include proteins, lipids, and nucleic acids, but cargo detection alone does not establish delivery to recipient cells or functional responsibility for a phenotype. Accordingly, this review evaluates EV-associated signalling as a plausible contributor to EMP, while calibrating mechanistic language to the controls and evidence reported in each study.

In ovarian cancer, malignant ascites is an EV-containing compartment in which particles from malignant and non-malignant cells coexist with abundant soluble factors. A frequently cited study reported an association between ascites-derived vesicle-associated miR-6780b-5p and EMT-related changes in ovarian cancer models [16]. These results support further investigation, but the degree to which the isolated material represented endosome-derived exosomes, and the relative contribution of vesicular versus soluble components, should be judged from the original study’s characterization and controls. The sections below therefore distinguish findings from patient samples, in vitro systems, and animal models rather than treating them as interchangeable evidence.

The conceptual contribution of this review is to integrate ovarian cancer EVs, EMT, and tumour microenvironment signalling around a specific question: when and how might EV-associated communication interact with reversible EMP states in the ascites-rich setting of HGSOC? We do not assume that every reported effect is exosome-specific or causal. Instead, we compare evidence by source and model, distinguish observed associations from functional perturbation and in vivo evidence, and identify where clinical validation is still lacking. This framework is intended to clarify the boundaries of current knowledge as well as the most actionable experimental gaps.

## 2. Biology of Extracellular Vesicles in Ovarian Cancer

### 2.1. Biogenesis

Exosomes are EVs formed through the endosomal pathway, in which intraluminal vesicles develop within multivesicular endosomes and are released when these compartments fuse with the plasma membrane [17,18]. Particle size alone (including the commonly reported 30–150 nm range) does not establish this biogenesis. In studies where endosomal origin has not been demonstrated, the isolated material is more accurately termed sEVs or an EV-enriched preparation. This distinction matters because commonly used isolation methods can co-isolate non-vesicular particles and soluble proteins.

Intraluminal vesicle formation within multivesicular endosomes can involve ESCRT-dependent and ESCRT-independent mechanisms, including pathways associated with ceramide and tetraspanins [19]. These mechanisms describe exosome biogenesis, but their presence should not be inferred solely from detection of EV markers. Claims that ovarian cancer cells preferentially release exosomes or that a specific biogenesis pathway drives progression require direct experimental support in the cited system [20,21,22].

### 2.2. Cargo Diversity

EV-associated RNA species, including microRNAs (miRNAs), long non-coding RNAs (lncRNAs), and circular RNAs (circRNAs), have been investigated in ovarian cancer. Studies report associations between selected RNA species in EV-enriched preparations and tumour cell phenotypes, including EMT-related marker changes, invasion, or drug response [20,21,23]. The strength of inference varies: enrichment in an isolated fraction does not by itself show that the RNA is protected within vesicles, delivered to recipient cells at a biologically relevant dose, or necessary for the observed phenotype. Where cargo manipulation, uptake controls, pathway perturbation, and rescue experiments are absent, these findings should be interpreted as associations or candidate mechanisms rather than established EV-mediated causality.

Long non-coding RNAs (lncRNAs; >200 nucleotides) and circular RNAs have also been reported in ovarian cancer EV-enriched preparations and investigated for possible roles in angiogenesis, metastasis, and chemoresistance [19,21]. Their proposed functions should be attributed to EV-associated fractions only when the original experiments distinguish vesicle-associated cargo from co-isolated material and demonstrate a functional effect in recipient cells.

**Figure 2:**
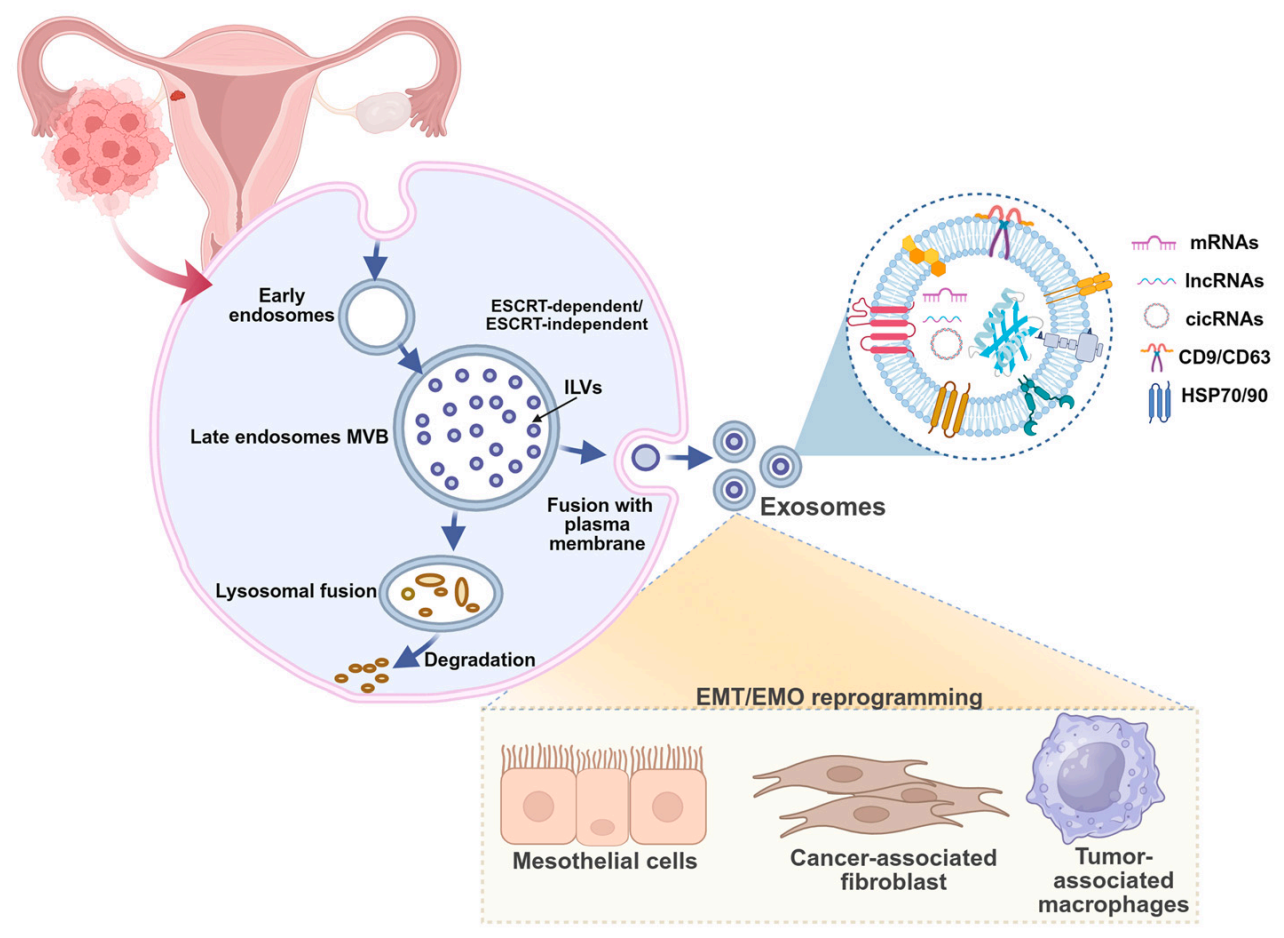
Schematic of endosomal exosome biogenesis and candidate EV cargo. The pathway shown represents established endosomal biology; it should not be taken to mean that every study cited in this review isolated endosome-derived exosomes. EV-associated cargo and downstream effects are context-dependent, and proposed links to EMT/EMP should be interpreted according to the experimental evidence available in each cited study. Figure 2 illustrates biogenesis and cargo loading; the broader cell-source and signalling network is discussed in Section 3 and Section 6 [25,26]. Created in BioRender. Ata Ullah. (2026) https://bit.ly/4sX48mH (accessed on 6 November 2026).

EV preparations contain membrane-associated proteins such as CD9, CD63, and CD81, as well as heat-shock proteins and other proteins and lipids [18,24]. These molecules can support characterization, but no single marker establishes endosomal origin or preparation purity. Claims that EV-associated proteins or lipids remodel extracellular matrix, angiogenesis, or metastasis should be linked to the specific experimental evidence and model in which those effects were demonstrated. Endosomal exosome biogenesis and the diversity of candidate EV cargo are summarized in Figure 2.

### 2.3. Sources of Extracellular Vesicles

EVs in the ovarian tumour microenvironment may originate from malignant cells and non-malignant populations, including fibroblasts, macrophages, mesothelial cells, and adipose-associated stromal cells (Figure 2) [25,26,27]. However, cellular source and vesicle subtype are not always resolved by the isolation methods used. Findings from other cancer types or simplified co-culture systems can provide mechanistic context but should not be presented as direct evidence for the same mechanism in human HGSOC unless validated in HGSOC patient material or relevant models.

Studies have reported that mesothelial cell-derived EV preparations promote ovarian cancer cell adhesion and invasion in peritoneal models [20]. Adipocyte-derived EV preparations can transfer lipids and metabolic regulators that support tumor growth and metastasis in the omental microenvironment [21]. EVs from cancer stem cell populations have also been reported to carry stemness-associated molecules linked to tumor heterogeneity, chemoresistance, and recurrence [23,28]. These cell sources and candidate cargo are illustrated in Figure 2.

## 3. Extracellular Vesicles as Inducers of EMT and EMP in Ovarian Cancer

EV-associated signalling has been investigated as one potential contributor to EMT-related changes and EMP in ovarian cancer. The evidence spans patient-sample associations, cell-culture experiments, and animal models, which answer different questions and should not be conflated. In this section, we summarize selected candidate mechanisms; Section 6 focuses on the broader ascites environment, and Section 7 addresses the steps of metastatic colonization to minimize repetition.

### 3.1. EV-Associated miRNAs Driving EMT

Among the most studied candidates is miR-6780b-5p, reported in ascites-derived vesicle preparations and associated with EMT-related changes and metastatic phenotypes in ovarian cancer models [16]. The reported functional effects are important, but claims of exosome-specific delivery require evidence that the preparation’s biogenesis was established, the cargo reached recipient cells, and the phenotype depended on that cargo rather than co-isolated material or other factors. The clinical association with disease severity is not, by itself, proof that this miRNA drives progression in patients.

miR-421 has been implicated in ovarian cancer cell-state regulation. In one study, exosome-associated miR-421 was reported to promote the formation of highly invasive ovarian cancer cell subsets through activation of the PI3K/AKT pathway [29]. These findings should not be interpreted as a universal mechanism: source, delivery route, recipient-cell context, and the strength of cargo-specific perturbation can influence biological outcomes. The miR-421 findings are discussed here only as an example of context-dependent candidate signalling; Section 5.3 provides the relevant heterogeneity context. 

### 3.2. Role of EV-Associated Proteins in Ovarian Cancer Progression

In addition to RNA cargo, EV preparations contain proteins that have been implicated in EMT-associated signaling, extracellular matrix remodeling, and metastatic behavior. Candidate proteins include TGF-β1, integrins, and matrix metalloproteinases (MMPs), each of which has been linked to invasion, adhesion, or cellular plasticity in ovarian cancer and other tumor systems [30,31,32]. However, the degree to which these effects are mediated specifically by endosome-derived exosomes varies among studies. Detection of a protein within an EV preparation does not establish functional delivery to recipient cells nor demonstrate that the cargo is necessary for the observed phenotype.

TGF-β signaling is among the best-characterized regulators of EMT-associated transcriptional programs and has been investigated in the context of EV-associated communication. Likewise, integrin- and MMP-associated pathways have been linked to cell adhesion, extracellular matrix remodeling, and metastatic dissemination. However, many studies do not distinguish vesicle-associated effects from those of co-isolated soluble factors, and relatively few directly establish cargo-specific causality through perturbation and rescue approaches.

Consequently, EV-associated proteins should be regarded as candidate mediators of cellular plasticity whose contribution to EMP depends on the quality of evidence available in individual experimental systems. Further studies are needed to determine which protein-mediated effects are attributable to defined EV populations in human HGSOC.

### 3.3. Key EV-Associated Signaling Networks in Ovarian Cancer

Several signaling pathways, including TGF-β/SMAD, PI3K/AKT, Wnt/β-catenin, Notch, NF-κB, and Hippo/YAP, have been associated with EMT-related phenotypes, cellular plasticity, and metastatic progression in ovarian cancer and other epithelial malignancies [33,34,35]. Their involvement in these processes does not establish that EVs initiate or regulate each pathway in HGSOC. Where studies do not directly perturb EV release, uptake, or candidate cargo while demonstrating pathway dependence, these pathways are more appropriately viewed as plausible downstream or parallel mechanisms rather than proven EV-mediated routes.

TGF-β/SMAD signaling remains one of the most extensively studied pathways linked to EMT-associated transcriptional programs, including regulation of SNAIL, ZEB, and TWIST family transcription factors [33,34]. Similarly, PI3K/AKT, Wnt/β-catenin, Notch, NF-κB, and Hippo/YAP signaling have each been associated with cell survival, invasion, stemness, and plasticity-related phenotypes in ovarian cancer [33,34,35]. In some experimental systems, EV-associated cargo has been proposed to influence these pathways; however, the extent to which pathway activation depends on specific EV populations, cargo molecules, or recipient-cell context remains incompletely defined.

Taken together, these signaling networks provide biologically plausible mechanisms through which EV-associated communication may influence EMP. Nevertheless, direct evidence establishing EV-specific regulation of these pathways in human HGSOC remains limited and warrants further investigation.

## 4. Hybrid Epithelial–Mesenchymal States: The Missing Link

### 4.1. What Is EMP?

EMT describes a set of coordinated changes through which epithelial cells acquire some mesenchymal features; mesenchymal-to-epithelial transition (MET) describes movement in the reverse direction. EMP is the broader capacity to move reversibly among epithelial, hybrid, and mesenchymal-like states. These terms are not interchangeable. Expression changes in one or two markers alone do not establish a complete EMT or MET, nor do they demonstrate a stable functional state. Stronger assignments combine multiple markers with morphology, transcriptional programmes, and relevant behaviors, interpreted in the experimental context [36,37,38,39].

### 4.2. Evidence in Ovarian Cancer

Ovarian cancer specimens and circulating tumor cell studies have reported heterogeneous epithelial and mesenchymal marker expression, consistent with possible hybrid states [38,39]. Such observations support the presence of phenotypic heterogeneity but do not, on their own, prove that individual cells transition dynamically or that EVs cause those states. Functional claims about spheroid formation, survival in ascites, or drug resistance require the relevant assays and should be distinguished from marker-based observations [39]. Evidence from non-HGSOC histotypes, cell lines, and other tumor types should be labelled as such rather than generalized to human HGSOC.

### 4.3. Hybrid E/M Phenotypes

Hybrid epithelial/mesenchymal states are most convincingly identified by coordinated evidence of epithelial and mesenchymal features in the same cells, ideally alongside functional assessment. Co-expression of E-cadherin or EpCAM with vimentin or EMT-associated transcription factors may be consistent with a hybrid state, but marker expression alone is insufficient to establish enhanced metastatic efficiency, stable identity, or reversibility [36,40]. Those functional properties depend on the model and should be described only where directly tested.

### 4.4. EV-Associated Control of Cell-State Transitions

EVs have been proposed to influence cell-state transitions through associated RNAs, proteins, and lipids [20,41]. The evidence is heterogeneous, and many studies do not establish endosomal origin or isolate the contribution of vesicles from soluble factors. Therefore, references to EV-mediated regulation of ZEB1, SNAIL, TGF-β, Wnt/β-catenin signaling [42], or a pro-plasticity niche should be read as candidate mechanisms unless supported by uptake evidence, cargo-specific perturbation, pathway inhibition, and appropriate rescue experiments. The principal unresolved issue is not simply whether EVs contain candidate cargo, but whether a defined EV population delivers functional cargo at relevant levels in HGSOC. Important gaps remain: the molecular determinants that stabilize hybrid states in vivo are incompletely defined; markers are not standardized across studies; and EV subtype, cargo delivery, and cell-of-origin are often unresolved. These limitations make it difficult to distinguish transient marker changes from stable or reversible EMP states and to assign a phenotype to a specific EV population [37,43].

## 5. Extracellular Vesicles and Intratumoral Heterogeneity

### 5.1. Clonal Evolution

HGSOC is characterized by pervasive chromosomal instability and near-universal *TP53* mutation, generating extensive subclonal genomic diversity within a single tumor mass [44]. Integrated single-cell whole-genome sequencing and spatial transcriptomics of treatment-naïve HGSOC reveal widespread copy-number diversification, concentrated in regions of copy-number gain, and show that whole-genome doubling (WGD) is a recurrent, ongoing evolutionary event rather than a single founder catastrophe that accelerates chromosomal loss and selects for highly adaptable clones; paradoxically, WGD-associated instability correlates with an immunosuppressive rather than immunostimulatory phenotype through repression of innate immune signaling [44,45]. Longitudinal clonal tracking combining single-cell whole-genome sequencing with time-series cell-free DNA shows that drug-resistant relapse typically arises from selective expansion of pre-existing clones rather than new genetic lineages, with resistant clones marked by chromothripsis, additional genome doubling, and amplification of oncogenes such as *CCNE1*, *MYC*, *RAB25*, and *NOTCH3*; clone-specific transcriptional programs, including EMT and VEGF signaling, pre-exist at diagnosis and are linked to eventual drug resistance, indicating that genetic and phenotypic evolution are tightly coupled in HGSOC [46]. High-plex spatial imaging integrating DNA-FISH with protein-level phenotyping further shows that subclonal copy-number heterogeneity is present even at the pre-invasive stage, with *MYC*- or *CCNE1*-amplified cells detectable in morphologically normal fallopian tube epithelium and serous tubal intraepithelial carcinoma, the putative HGSOC precursor lesion [47]. HGSOC therefore accumulates clonal diversity long before clinically apparent disease, providing the principal substrate for selection of resistant and metastasis-competent subpopulations under therapeutic pressure [44,46].

### 5.2. Non-Genetic Heterogeneity

Genetic subclonal architecture alone does not fully account for the phenotypic diversity observed within ovarian tumors. Single-cell transcriptomic profiling of HGSOC reveals extensive cell-state heterogeneity, including coexisting epithelial-like and mesenchymal-like transcriptional programs, immune-evasive subpopulations, and stemness-associated clusters not strictly predicted by underlying copy-number or mutational status [12]. Comparable single-cell atlases across ovarian tumor, ascites, and metastatic sites have identified recurrent malignant cell states—including a metastasis-associated cluster marked by RAB13 overexpression—that vary in abundance across anatomical niches independently of clonal lineage, implying that the tumor microenvironment itself sculpts transcriptional identity [48]. More broadly, work in other epithelial malignancies demonstrates that tumor cells occupy a spectrum of epithelial and mesenchymal-like transcriptional states and transition bidirectionally between them, both within and across genetically defined subclones, driven by regulators such as SMAD3, KLF4, and PPARG rather than discrete mutational events; this non-genetic, dynamically reversible heterogeneity likely arises from stochastic gene-expression noise, chromatin-state variability, and microenvironmental signaling, and can be selected for and against under stress such as nutrient limitation or cytotoxic therapy, reconstituting population-level heterogeneity even after a bottleneck [49]. Conceptual frameworks describing drivers of dynamic intratumor heterogeneity emphasize that non-genetic plasticity operating on timescales of hours to days rather than the many cell divisions required for clonal selection—allows tumors to sample a broader range of adaptive states than genetic diversification alone, underlying the coexistence of epithelial, mesenchymal, and hybrid E/M states within the same tumor and providing a reservoir of phenotypic flexibility mobilized during metastatic dissemination and chemotherapy [12,50].

### 5.3. EV-Associated Reprogramming

EV-associated cargo exchange may contribute to phenotypic differences between ovarian cancer subpopulations. For example, one cell-line study reported that vesicle preparations from highly invasive subpopulations were enriched in miR-328-3p and altered migration/invasion-related phenotypes in less-invasive cells [51]. Separately, omental-explant-conditioned material was reported to contain miR-421 associated with CBX7 regulation [52]. These are mechanistic leads from experimental systems, not proof that the same pathways operate in patients. For miR-421, the CBX7 study is the principal example discussed here; Section 3 briefly contextualizes its reported effects, while other sections avoid repeating the mechanism.

## 6. The Ascites Ecosystem as a Plasticity Niche

### 6.1. Composition of Malignant Ascites

Malignant ascites develops in most patients with advanced ovarian cancer and constitutes a distinctive liquid tumor microenvironment rather than a passive transudate [5]. Its cellular compartment includes exfoliated tumor cells surviving as single cells, multicellular spheroids, or hybrid aggregates; mesothelial cells shed from the peritoneal lining, some undergoing mesothelial–mesenchymal transition and adopting cancer-associated fibroblast-like functions; tumor-associated macrophages recruited from circulating monocytes and frequently skewed toward an M2-like polarization state; lymphocytes; and adipocyte-derived and mesenchymal stromal populations contributed by the omentum [5,53]. These macrophages correlate with clinical outcome: surface expression of the M2 marker CD163 is associated with shorter relapse-free survival and correlates with ascites levels of IL-6 and IL-10, both of which independently induce CD163 expression, indicating a self-reinforcing immunosuppressive circuit [54]. The acellular fraction is equally consequential, comprising cytokines, chemokines, and growth factors—including IL-6, interleukin-8, VEGF, lysophosphatidic acid, and TGF-β—together with complement components, coagulation factors, and extracellular matrix proteins such as fibronectin and hyaluronan; IL-6 functions as a central node of this network, promoting tumor cell proliferation, angiogenesis, and metastatic dissemination while reinforcing macrophage polarization and STAT3 activation [55,56]. This combination of exfoliated tumor and stromal cells within a cytokine-rich fluid distinguishes malignant ascites from a simple byproduct of impaired lymphatic drainage, positioning it as an active, tumor-supportive ecosystem [5,55].

### 6.2. Ascites-Derived Extracellular Vesicles

**Figure 3:**
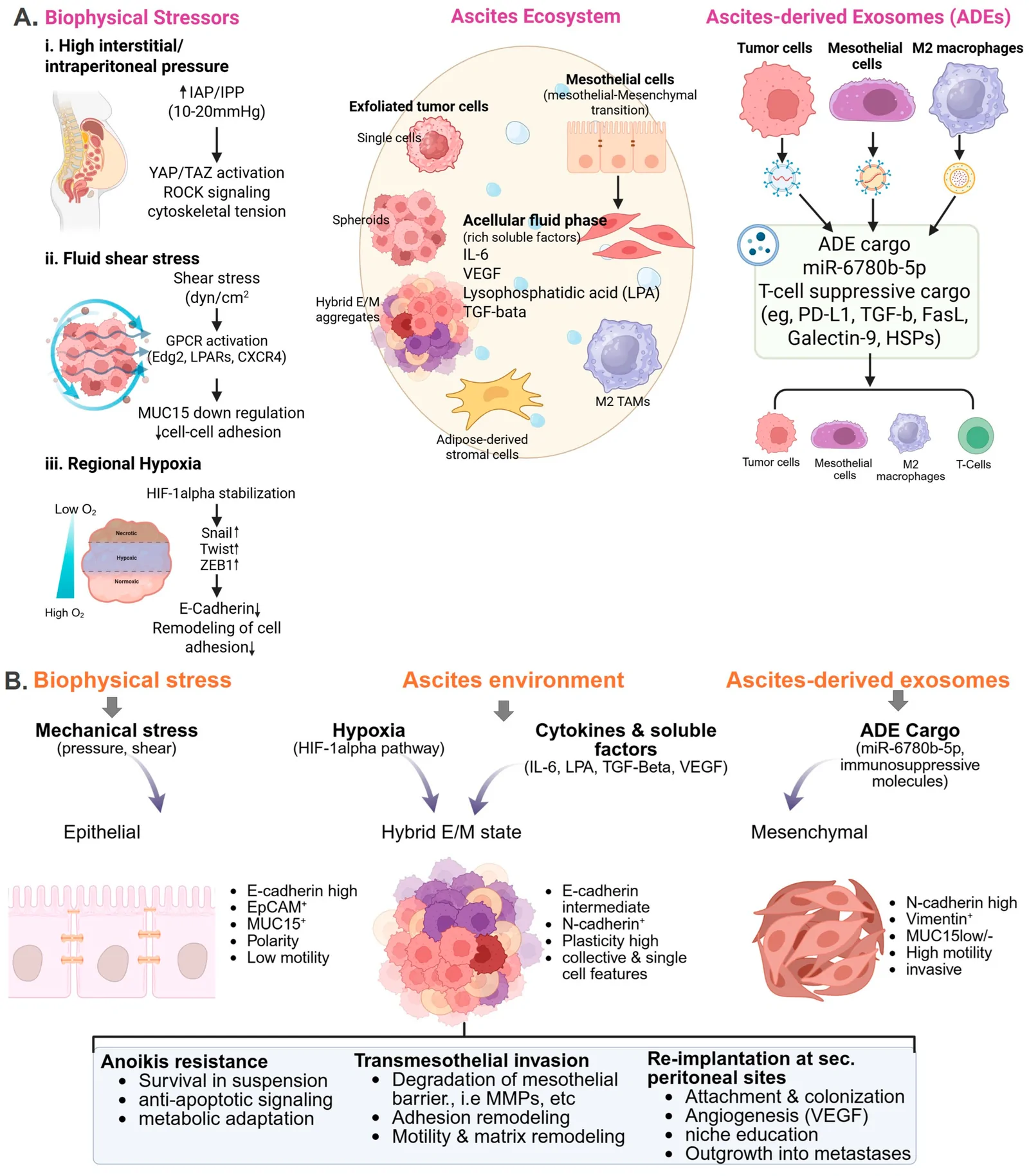
Proposed interactions within the ascites ecosystem that may promote epithelial–mesenchymal plasticity. (**A**) Schematic overview of the malignant ascites microenvironment and factors associated with epithelial–mesenchymal plasticity (EMP) in disseminated ovarian cancer cells. The panel depicts the cellular composition of ascites, including exfoliated tumor cells (single cells, multicellular spheroids, and hybrid E/M aggregates), mesothelial cells undergoing mesothelial-mesenchymal transition, M2-polarized tumor-associated macrophages (TAMs), and adipose-derived stromal cells, suspended within an acellular fluid phase enriched in IL-6, VEGF, lysophosphatidic acid, and TGF-β [5,53,56]. The panel further illustrates biophysical stressors acting on suspended cells, including elevated intra-abdominal pressure (IAP) and intraperitoneal pressure (IPP), fluid shear stress, and regional hypoxia, together with representative molecular responses. Ascites-derived exosomes (ADEs) originating from tumor, mesothelial, and macrophage sources are shown carrying miR-6780b-5p and immunomodulatory cargo between cellular compartments [16,65]. (**B**) Conceptual model illustrating how mechanical stress, hypoxia, cytokine signaling, and EV-associated cargo may contribute to transitions along an epithelial-to-hybrid epithelial/mesenchymal (E/M)-to-mesenchymal continuum. These transitions are associated with phenotypes linked to anoikis resistance, transmesothelial invasion, and re-implantation at secondary peritoneal sites in experimental models [66,67]. The figure represents a conceptual synthesis of findings from multiple experimental systems and should not be interpreted as a single validated mechanism established in patients. Created in BioRender. Ata Ullah. (2026) https://bit.ly/4sX48mH (accessed on 6 November 2026).

Ascites-derived EV preparations are likely heterogeneous and may include material from malignant cells, mesothelial cells, macrophages, and adipose-associated stroma, together with soluble proteins and other extracellular particles (Figure 3) [57,58]. A study reported an association between miR-6780b-5p in an ascites-derived preparation and EMT-related effects in ovarian cancer models [16]. Unless endosomal origin, cargo localization, uptake, and cargo dependence were established, the preparation should be described as ascites-derived EVs/sEVs rather than definitively as exosomes. Ascites heterogeneity limits attribution of observed effects to a single vesicle subtype or cellular source.

### 6.3. Mechanical Stress and Ascites

Malignant ascites exposes tumor cells to biochemical and physical conditions that may influence survival and phenotype. Malignant ascites exposes tumor cells to biochemical and physical conditions, including elevated interstitial stress and fluid pressure, that may influence survival and phenotype [59]. Experimental studies of shear stress, pressure, hypoxia, and acidity have reported changes in adhesion, cytoskeletal organization, or EMT-associated markers in particular models (Figure 3) [60,61,62,63]. In ovarian cancer models, hypoxia and IL-6 signaling have also been reported to act synergistically through a STAT3/HIF-1α feedback loop that promotes EMT-associated invasion [64]. These findings do not establish that EVs mediate the effects of each stressor, nor do in vitro exposure systems fully reproduce patient ascites dynamics. We therefore treat these factors as complementary environmental pressures and distinguish model-based mechanisms from evidence directly demonstrated in patients. Figure 3 integrates these cellular, soluble, and biophysical features of the ascites ecosystem with potential EMP-associated responses.

### 6.4. Ascites-Induced EMP

Studies exposing ovarian cancer cell lines to patient-derived ascites have reported changes in morphology and EMT-associated markers, and follow-up experiments have implicated soluble growth factors and downstream signalling in some systems [66,68]. Other work describes spheroid-associated hybrid phenotypes and reversible marker changes after reattachment, as shown in Figure 3 [67,69]. These results support a dynamic, context-dependent model of cell-state adaptation, but marker shifts should not automatically be equated with complete EMT or stable EMP. Because ascites contains EVs, soluble mediators, cells, and other particles, effects of whole ascites cannot be attributed specifically to EVs without fractionation, depletion/add-back, uptake, and cargo-dependence controls. Clinical relevance should be framed as plausible unless directly validated in patient cohorts.

## 7. Extracellular Vesicles and Metastatic Colonization

### 7.1. Peritoneal Dissemination

Unlike most solid tumors, HGSOC rarely metastasizes hematogenously in its early phases; instead, it disseminates predominantly through transcoelomic spread, in which cells or clusters shed from the primary tumor are transported passively by peritoneal fluid to seed serosal surfaces throughout the abdominal cavity [70]. This depends on the tumor’s ability to survive in a matrix-detached, anchorage-independent state, normally lethal to epithelial cells via anoikis [71]. Ovarian cancer cells circumvent this checkpoint through integrin-mediated survival signaling and re-expression of basement-membrane components; Notch3 overexpression drives anoikis resistance by inducing COL4A2 and sustaining FAK–AKT–ERK survival signaling while repressing the pro-apoptotic protein Bim [72]. This resistance is itself an acquired, reversible state: repeated detachment/re-attachment cycles select for a phenotype that also confers enhanced invasiveness, chemoresistance, and immune evasion, sensitive to pharmacological CDK8/19 Mediator kinase inhibition [73].

A defining histological feature of ovarian cancer ascites is the multicellular spheroid, formed as exfoliated cells self-associate in suspension [74]. Compact, E-cadherin-junction-linked architecture protects interior cells from anoikis and confers chemoresistance by limiting drug penetration; spheroids contacting the mesothelium flatten and disaggregate, allowing peripheral cells to invade while a compacted core remains available for further transport [74]. Ascites-derived EVs may reinforce this ecosystem: vesicle preparations from ovarian cancer ascites have been reported to enhance tumor cell proliferation and migration, suggesting that the ascites-borne vesicular compartment may support spheroid viability and invasive competence [19,75].

### 7.2. Mesothelial Reprogramming

The mesothelium lining the peritoneal cavity represents the first barrier encountered by disseminated ovarian cancer cells during transcoelomic spread [70]. Experimental evidence suggests that EV-associated signaling may contribute to mesothelial reprogramming and facilitate subsequent tumor cell adhesion and invasion. However, the relative contribution of EVs compared with soluble mediators and direct cell–cell interactions remains incompletely resolved.

Several studies have reported uptake of tumor-derived EVs by human peritoneal mesothelial cells (HPMCs), accompanied by morphological and transcriptional changes consistent with mesothelial-to-mesenchymal transition (MMT) [76]. Proposed mechanisms include EV-associated transfer of CD44, miR-99a-5p, TGF-β1, and other candidate mediators that may alter extracellular matrix composition, cellular adhesion, and stromal activation [70,76,77]. Although these findings support a role for EV-associated communication, the strength of evidence varies among studies, and causal relationships should be interpreted in the context of the underlying experimental design.

Collectively, these observations suggest that EV-associated signaling may participate in remodeling the mesothelial barrier and creating a more permissive microenvironment for implantation and invasion. Nevertheless, further work is required to define the contribution of specific EV populations and cargo molecules in human HGSOC.

### 7.3. Omental Colonization

The greater omentum is the single most frequent site of ovarian cancer metastasis, with disseminated cells localizing there within one to six hours of entering the peritoneal cavity in experimental models [78]. This tropism follows a two-step model in which milky spots, immune aggregates of macrophages, lymphocytes, and stromal cells around glomerulus-like capillary tufts, mediate initial lodging, after which surrounding adipocytes support outgrowth; conditioned medium from milky-spot-containing tissue increases migration by roughly 75% relative to milky-spot-deficient fat [79]. Omentum-resident CD163+Tim4+ macrophages of embryonic origin are indispensable: paracrine JAK–STAT3 signaling, likely via IL-6, erythropoietin, and prolactin, drives EMT and cancer stem cell (CSC) marker acquisition, and depletion of this subset abrogates metastatic outgrowth [80]. Complementary CCL6/CCL23–CCR1 chemokine signaling further enhances colonization, and elevated tumoral CCR1 independently predicts shorter progression-free survival [78]. Once lodged, tumor cells engage in metabolic crosstalk with omental adipocytes: adipocytes transfer free fatty acids that are beta-oxidized by tumor cells, accelerating proliferation, while omental adipocyte content inversely tracks metastatic burden as fat that is depleted by the expanding tumor [81]. Cancer-associated adipocytes also adopt a secretory phenotype reinforcing chemoresistance; adipocyte/CAF-derived EV-associated miR-21 suppresses tumor cell apoptosis via APAF1 downregulation [70]. Collectively, these observations support the view that the omentum provides a particularly supportive environment for metastatic colonization in experimental ovarian cancer models.

### 7.4. Premetastatic Niche Formation

Experimental studies in multiple tumor systems suggest that tumor-derived EVs may contribute to premetastatic niche formation by influencing stromal, immune, and vascular compartments before overt metastatic colonization [82]. Whether identical mechanisms operate in ovarian cancer, and to what extent they contribute to human disease progression, remains an active area of investigation.

Proposed mechanisms include EV-mediated modulation of inflammatory signaling, extracellular matrix remodeling, vascular permeability, and recruitment of supportive stromal or immune populations [82,83]. Integrin-associated EV uptake has been implicated in organ-specific tropism in experimental models, while ovarian cancer studies have reported that EV-associated cargo such as miR-141 may influence fibroblast behavior and stromal signaling pathways linked to metastatic competence [83,84].

These findings support the hypothesis that EV-associated communication may contribute to establishment of permissive metastatic microenvironments. However, much of the available evidence originates from animal models or experimental systems, and direct validation in human HGSOC remains limited. Accordingly, premetastatic niche formation should be viewed as a promising mechanistic framework rather than a clinically established process directed by ovarian cancer EVs.

## 8. EV-Associated Therapeutic Resistance

### 8.1. Platinum Resistance

Platinum-based chemotherapy remains the backbone of first-line ovarian cancer treatment, yet resistance develops in most patients with advanced disease. Increasing evidence suggests that tumor microenvironmental factors, including EV-associated signaling, may contribute to therapeutic persistence, dormancy, and relapse [85]. EV preparations from platinum-resistant cell lines have been reported to transfer resistance-associated phenotypes to sensitive recipients, together with EMT-like changes such as reduced E-cadherin/dystroglycan and increased vimentin/TWIST [86]. In the cited study, resistant-cell EV preparations also increased mature miR-21 in recipient cells nearly four-fold through enhanced processing rather than transcription, raising the carboplatin IC50 by up to seven-fold [86]. A separate study reported that DNMT1-enriched EV preparations from cisplatin-resistant cells stimulated endogenous DNMT1 in recipient cells and conferred resistance in vitro and in xenografts; this effect was reversed by the EV-release inhibitor GW4869 [87].

**Figure 4:**
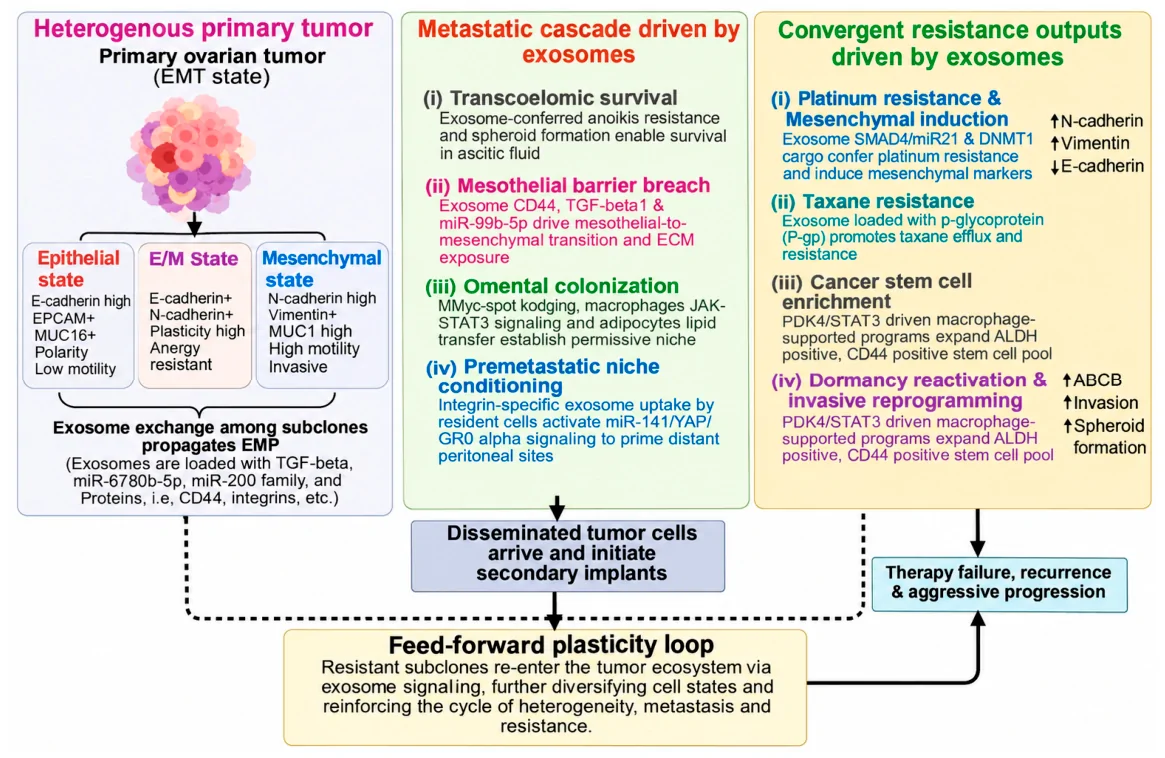
Conceptual model linking EV-associated signaling, epithelial–mesenchymal plasticity, intratumoral heterogeneity, metastatic colonization, and therapeutic resistance in ovarian cancer. The left panel depicts a heterogeneous primary tumor containing epithelial, hybrid E/M, and mesenchymal-like cell states, with EV-associated communication proposed as one mechanism that may contribute to phenotypic plasticity among tumor cell subpopulations. The central panel summarizes processes associated with the metastatic cascade, including anoikis resistance and spheroid formation during transcoelomic dissemination [73,74], mesothelial remodeling linked to EV-associated CD44, TGF-β1, and miR-99a-5p signaling [76,77], omental colonization supported by macrophage-derived and adipocyte-derived cues [79,80], and stromal conditioning mediated in part by EV-associated signaling pathways implicated in premetastatic niche formation [83,84]. The right panel summarizes mechanisms associated with therapeutic resistance, including EV-associated transfer of factors linked to platinum resistance [86,87], taxane resistance [89], cancer stem cell enrichment [80,93], and dormancy/reactivation phenotypes [95]. Feed-forward arrows illustrate the proposed interplay between cellular plasticity, metastatic competence, and therapeutic resistance, whereby resistant or reactivated cell populations may further contribute to tumor heterogeneity. This figure represents a conceptual synthesis of findings from multiple experimental systems and should not be interpreted as a single validated causal mechanism or as evidence that EV-associated signaling alone is sufficient to drive all stages of metastatic progression and treatment resistance. Created in BioRender. Ata Ullah. (2026) https://bit.ly/4sX48mH (accessed on 6 November 2026).

Another proposed mechanism is the export of cisplatin in EVs: resistant cells were reported to release approximately 2.6-fold more vesicle-associated platinum than sensitive cells, lowering intracellular drug concentrations below cytotoxic thresholds [15]. Hypoxia (Figure 4) may amplify this process through STAT3-dependent Rab27a upregulation and Rab7/LAMP1/2 downregulation, increasing EV release two- to six-fold; EV preparations from hypoxic cells carried approximately five-fold more cisplatin, together with oncogenic STAT3 and FAS, and conferred chemoresistance on recipient cells [88]. CAF-derived EV-associated miR-98-5p has likewise been reported to increase cisplatin resistance through CDKN1A targeting, suggesting that resistance can propagate through a stromal, multicellular EV network [70].

### 8.2. Taxane Resistance

Resistance to paclitaxel, platinum’s companion agent, can also involve vesicle-mediated transfer of the efflux pump P-glycoprotein (P-gp/ABCB1). Paclitaxel-resistant A2780 sublines shed markedly more P-gp-containing microvesicles than sensitive counterparts, which bind and transfer P-gp into recipients time- and dose-dependently, redistributing co-administered drugs away from intracellular targets; recipients acquire roughly five-fold higher resistance to both paclitaxel and the structurally distinct adriamycin, indicating a broad multidrug-resistant phenotype [89]. This explains how efflux-transporter overexpression spreads through a heterogeneous population faster than clonal selection alone predicts, since resistance-conferring cargo is actively disseminated to naive cells [90]. EV-associated microRNAs, including miR-1246, form a complementary axis, modulating apoptotic/survival signaling to blunt cytotoxicity in parallel with the ABC-transporter mechanism [15], mirroring platinum-resistance programs and indicating mechanistic overlap across drug classes [91].

### 8.3. Cancer Stem Cell Enrichment

A subpopulation of ovarian cancer cells with stem-like properties, self-renewal, tumor-initiating capacity, and elevated CD44/CD133/ALDH1, is enriched in ascites and strongly linked to chemoresistance and recurrence [92]. Ascites-derived ALDH+CD44+ subsets show enhanced sphere-forming capacity and a PDK4-mediated glycolytic switch that drives STAT3/AKT/NF-κB/IL-8 signaling to sustain OCT4, KLF4, and BMI1; PDK4 blockade suppresses these properties and sensitizes cells to cisplatin [93]. EVs may propagate this compartment beyond cell-intrinsic origins: EV-associated miR-454 sustains stemness via the PRRT2/Wnt axis in recipients that would not otherwise display a stem phenotype [94]. Wnt/β-catenin, a canonical driver of stemness and EMT alike, promotes proliferation, migration, invasion, and therapy resistance in ovarian cancer [42]. Omentum-resident CD163+Tim4+ macrophages amplify this axis via JAK–STAT3-driven simultaneous EMT and CSC marker acquisition, and depletion abrogates both invasive spread and stemness [80]. This is clinically consequential, since ovarian CSCs resist chemotherapy owing to elevated efflux transporters, enhanced DNA repair, and quiescence, so EV-driven CSC expansion amplifies chemoresistance population-wide, even absent additional resistance cargo [92]. Targeting the EV-associated machinery sustaining CSC plasticity, rather than the stem cells themselves, has been proposed to prevent reservoir replenishment after therapy [15].

### 8.4. Dormancy and Recurrence

Ovarian cancer recurrence, affecting most advanced-stage patients despite optimal debulking and platinum-taxane chemotherapy, is increasingly attributed to dormant cells that survive treatment before reactivation drives relapse [95]. The tumor microenvironment and its EV output actively regulate this transition, holding cells dormant or triggering reawakening [85]. A recent study implicates chemotherapy itself via a senescence-EV relay: carboplatin/paclitaxel exposure drives peritoneal mesothelial cells and fibroblasts into iatrogenic senescence, and these cells secrete markedly more EVs than non-senescent counterparts (Figure 4) [95]. EVs from senescent mesothelial cells, carrying reduced miR-210-3p/miR-409-3p/miR-421 and elevated MMP1/MMP3/VEGF, and EVs from senescent fibroblasts, enriched in amphiregulin and osteopontin, both reactivate dormant cells more efficiently than EVs from non-senescent cells, promoting migration, invasion, and spheroid formation while inducing CCL11 and ABCB1 [95]. This indicates chemotherapy given to eliminate residual disease can, via stromal senescence, generate a secondary signal that seeds recurrence. EV-associated integrins implicated in premetastatic niche formation (Section 7.4) also help maintain dormant disseminated cells at distant sites via ITGB6 and related adhesion signaling [85]. EV transfer from mesenchymal stromal cells can conversely suppress proliferative/EMT signaling, inducing rather than reversing dormancy, indicating this regulation is bidirectional and context-dependent (Figure 4) [96]. These findings position EV traffic between residual tumor cells and treatment-altered stroma as a key determinant of latency between remission and recurrence, and a potential therapeutic target.

### 8.5. EMP-Resistance Axis

Collectively, the studies discussed above support the hypothesis that mesenchymal, hybrid epithelial/mesenchymal, and stem-like states may contribute to therapeutic resistance in ovarian cancer. Several signaling pathways implicated in EMP, including TGF-β/SMAD, PI3K/AKT, Wnt/β-catenin, and STAT3, have also been associated with resistance to platinum- and taxane-based therapies [86,93,96,97]. This overlap has led to the proposal that cellular plasticity may provide a common framework linking metastatic competence and treatment failure.

Evidence from experimental models indicates that EV-associated cargo can induce changes in EMT-associated markers, stemness programs, and drug-response phenotypes [86,87,88,89,90,91,92,93,94,95]. However, these observations should not be interpreted as definitive proof that EMP directly causes therapeutic resistance in human HGSOC. Many reported mechanisms derive from cell-culture systems or animal models, and the relative contributions of EV-associated signaling, genetic evolution, and broader microenvironmental influences remain difficult to disentangle.

A central concept emerging from these studies is that cellular plasticity may allow tumor cells to shift between phenotypic states that favor dissemination, survival under therapeutic stress, dormancy, and eventual recurrence. Within this framework, EV-associated communication represents one potential mechanism capable of influencing these transitions. Whether specific EV populations are necessary or sufficient to drive durable EMP-associated resistance in patients remains unresolved.

Taken together, current evidence supports a model in which EV-associated signaling, EMP, intratumoral heterogeneity, metastasis, and therapeutic resistance are interconnected processes. However, additional mechanistic studies and clinical validation are necessary before these relationships can be considered causally established or therapeutically actionable.

## 9. Emerging Therapeutic Strategies

### 9.1. Inhibiting EV Biogenesis and Release

Evidence that EV-associated signalling may contribute to EMP, immune evasion, and chemoresistance has motivated strategies to reduce vesicle biogenesis and release. GW4869, a non-competitive inhibitor of neutral sphingomyelinase-2 (nSMase2), reduces ceramide-dependent intraluminal vesicle formation and EV release but does not selectively inhibit a biogenetically confirmed exosome population [98,99]. In prostate cancer models, GW4869 reduced EV-associated immune dysfunction and reversed CD8+ T-cell exhaustion [100]. In small-cell lung cancer models, combined nSMase2/RAB27A inhibition with GW4869 and Nexinhib20 reduced CD9, CD63, and TSG101 in EV preparations and enhanced cisplatin/etoposide cytotoxicity [101]. GW4869 has also reduced EV-associated paracrine effects such as adipose browning in cancer-associated cachexia models [102]. These findings from non-ovarian systems provide mechanistic rationale but do not establish therapeutic efficacy in ovarian cancer.

**Figure 5:**
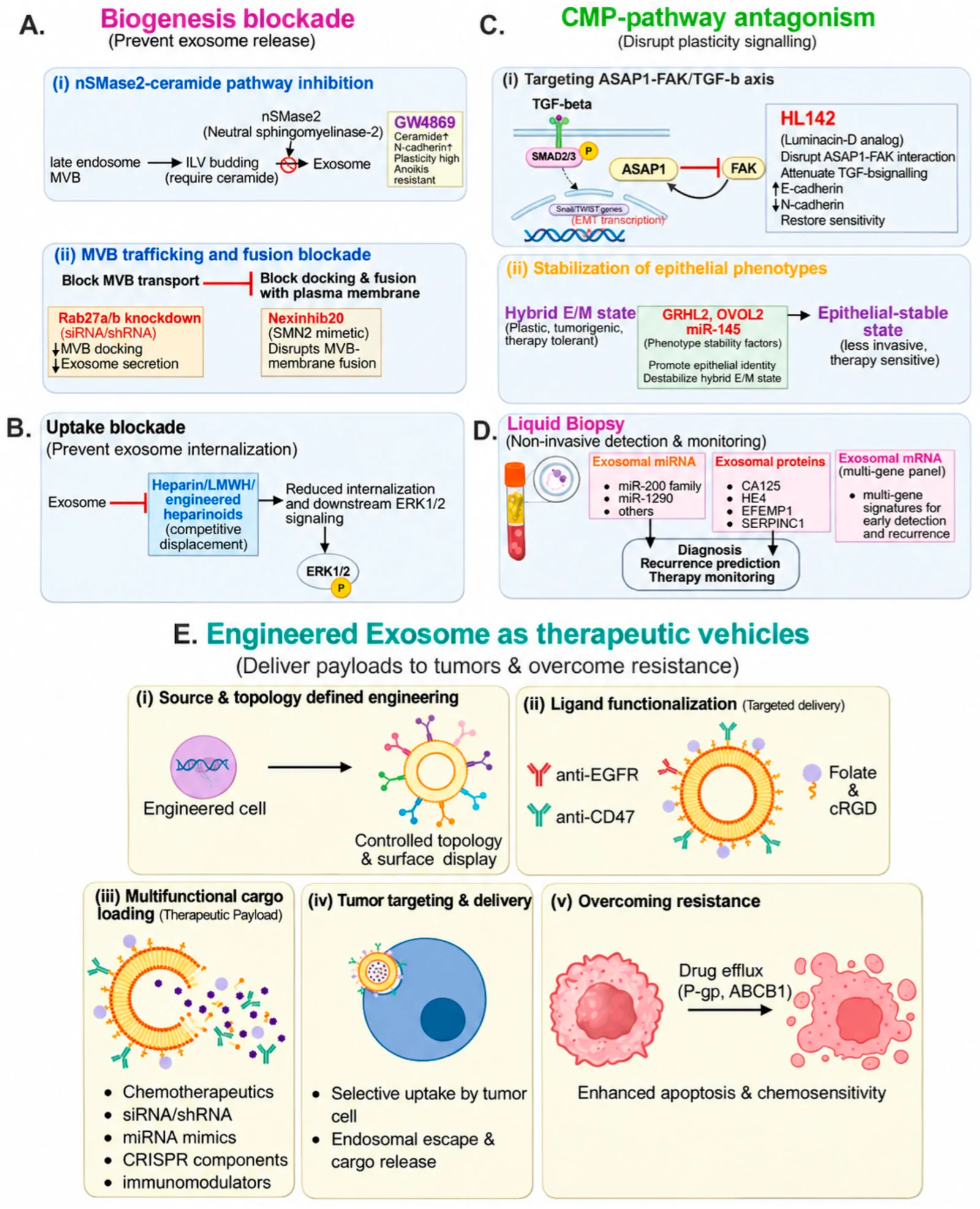
Therapeutic targeting and engineered EV strategies. Schematic overview of proposed intervention points within EV-associated pathways relevant to ovarian cancer. (**A**) Biogenesis blockade: GW4869 inhibits neutral sphingomyelinase-2 (nSMase2)-dependent ceramide generation required for intraluminal vesicle budding [98], while Rab27a/b knockdown or Nexinhib20 disrupts multivesicular body docking and plasma membrane fusion [101,103]. (**B**) Uptake blockade: heparin, low-molecular-weight heparin, and engineered heparinoids competitively interfere with EV interactions with cell-surface heparan sulfate proteoglycans, reducing internalization and downstream signaling in recipient cells [107]. (**C**) EMP-pathway antagonism: small molecules such as the luminacin D analog HL142 disrupt ASAP1-FAK interactions and attenuate TGF-β signaling, restoring epithelial marker expression and chemosensitivity [109], while phenotypic stability factors (GRHL2, OVOL2, miR-145) have been proposed as candidate regulators of hybrid E/M states [111]. (**D**) Liquid biopsy: circulating EV-associated miRNA, protein, and mRNA signatures are being investigated as potential biomarkers for ovarian cancer diagnosis, prognosis, recurrence monitoring, and treatment response [114,115,116]. (**E**) Engineered EVs: surface engineering, ligand functionalization (folate, anti-EGFR, cRGD, CD47), and therapeutic cargo loading (chemotherapeutics, siRNA, and miRNA mimics) are being explored as approaches to improve targeted delivery and overcome resistance mechanisms in preclinical studies, with some EV-based technologies undergoing early clinical evaluation [117,118,119]. Arrows indicate potential points at which these strategies may interfere with EV-associated signaling pathways implicated in EMP, metastasis, and therapeutic resistance. This figure represents a conceptual summary of investigational approaches and should not be interpreted as evidence of clinically established therapeutic benefit in ovarian cancer. Created in BioRender. Ata Ullah. (2026) https://bit.ly/4sX48mH (accessed on 6 November 2026).

A parallel strategy targets the Rab GTPase machinery governing multivesicular body docking and fusion with the plasma membrane. Rab27a and Rab27b act at discrete steps of the secretory pathway—Rab27a controlling MVB size and distribution, Rab27b regulating perinuclear-to-peripheral trafficking—and silencing either GTPase, or their effectors Slp4 and Slac2b, suppresses EV release (Figure 5) [103]. In melanoma, RAB27A knockdown/knockout reduces secretion of a pro-invasive EV subpopulation and diminishes invasion and metastasis in vivo, though with compensatory shifts in vesicle subpopulation composition, a caveat for single-node EV-release blockade strategies [104]. Elevated Rab27a/b expression correlates with metastasis and poor survival across solid tumors, reinforcing the rationale for Rab27 blockade in ovarian cancer, where EMP and peritoneal dissemination are central to disease biology [103]. Other blockers under investigation include ESCRT-machinery disruption and Rab35, which similarly regulates MVB docking [105]. Collectively, strategies targeting EV biogenesis, uptake, EMP-associated signaling pathways, biomarker development, and engineered EV delivery systems are summarized in Figure 5.

### 9.2. Blocking EV Uptake

EV uptake can also be reduced by interfering with recipient-cell internalization. Cell-surface heparan sulfate proteoglycans (HSPGs) serve as principal internalizing receptors for cancer-derived EVs, and competitive displacement with soluble heparan sulfate, heparin, or low-molecular-weight heparins reduces uptake in a dose-, size-, and charge-dependent manner, attenuating downstream ERK1/2 activation and EV-stimulated migration; enzymatic removal of surface HSPGs produces comparable effects, implicating HSPG-dependent internalization as a druggable checkpoint (Figure 5) [106]. Structure–activity analyses show 6-O-sulfation is critical for EV formation and functional transfer, with desulfated heparin losing much of the inhibitory capacity of fully sulfated heparin, indicating engineered heparinoids—rather than clinical anticoagulant heparin per se—may be optimal uptake blockers [107]. Heparinoids lacking anti-factor Xa activity may retain EV-inhibitory activity while reducing bleeding risk, a potentially useful property for ovarian cancer patients who already have an elevated thromboembolic risk [108].

### 9.3. Targeting EMP

Because EMP integrates converging signaling inputs rather than a single linear pathway, strategies have largely focused on destabilizing the transcriptional circuits (TGF-β, FAK/ASAP1, SNAIL/ZEB) maintaining the mesenchymal or hybrid state, rather than EV inhibition alone. The luminacin D analog HL142 disrupts the ASAP1–FAK interaction, attenuates TGF-β/FAK signaling, upregulates E-cadherin and cytokeratin-7, downregulates vimentin, β-catenin, and SNAIL2, and suppresses tumor growth and peritoneal metastasis in orthotopic ovarian cancer models while sensitizing cells to paclitaxel [109]. Broader reviews of EMT/cancer stem cell-directed therapy catalogue additional small molecules capable of resensitizing chemoresistant ovarian tumor cells by disrupting SNAIL/SLUG transcription factor networks (Figure 5) [110]. Mathematical modeling of the EMT regulatory network indicates phenotypic stability factors (PSFs) such as GRHL2, OVOL2, and miR-145 can be manipulated to destabilize the hybrid E/M state, offering a route to pharmacologically “push” plastic tumor cells toward a fixed, less invasive phenotype [111,112]. Because EV-associated cargo may contribute to EMP induction in recipient cells within the ascites and stroma, EV biogenesis/uptake blockade (Section 9.1 and Section 9.2) and direct EMP-pathway antagonism are increasingly viewed as complementary strategies [113].

### 9.4. Liquid Biopsy Approaches

EV-associated molecules are being explored as minimally invasive biomarkers for diagnosis, prognosis, recurrence monitoring, and treatment response (Figure 5). At present, candidate discovery and biological plausibility should be distinguished from analytical validation and demonstrated clinical utility. Claims of clinical readiness require reproducibility, independent validation cohorts, prespecified performance metrics, and evidence of added value over established clinical measures. Where studies report only associations or small exploratory cohorts, describe the findings as preliminary candidates rather than validated biomarkers. Figure 5 provides a schematic overview of proposed EV-targeting interventions, engineered EV strategies, and related translational approaches.

### 9.5. Engineered Extracellular Vesicles

Engineered EVs and strategies to inhibit EV release or uptake remain at different stages of preclinical and translational development. Results in cell culture or animal models do not establish clinical efficacy, safety, manufacturing feasibility, or tumour-selective delivery. Clinical-trial statements must be checked against the live registry record and described according to the actual study design and status (Figure 5). Although engineered EVs have demonstrated promise as delivery vehicles for therapeutic cargo in preclinical cancer models, their clinical translation remains at an early stage. Key challenges include manufacturing standardization, biodistribution, cargo-loading efficiency, safety, immunogenicity, and large-scale production. Ongoing clinical studies are evaluating EV-based technologies for biomarker development and disease monitoring, but evidence supporting therapeutic efficacy in ovarian cancer remains limited. Therefore, engineered EV approaches should currently be viewed as promising investigational strategies rather than clinically established therapies. A summary of the major therapeutic intervention points discussed throughout this section is provided in Figure 5.

## 10. Knowledge Gaps and Future Directions

The evidence base has several limitations. First, many reports use EV-enriched preparations without demonstrating endosomal biogenesis, and isolation methods may co-enrich soluble proteins, lipoproteins, or other particles. Future studies should follow MISEV2023 recommendations, report source material and isolation/characterization methods, and use orthogonal characterization and appropriate negative controls [120]. Second, causal claims require evidence beyond cargo enrichment: uptake and delivery, cargo manipulation, pathway perturbation, and rescue experiments are important where feasible. Third, studies should distinguish patient-derived HGSOC evidence from other ovarian histotypes, non-ovarian cancers, cell lines, and animal models; model limitations include genotype, treatment history, clonal composition, and microenvironmental complexity.

A second priority is to define EMP with coordinated molecular and functional measurements. EMT, MET, and hybrid states should be distinguished, and isolated marker changes should not be interpreted as proof of a complete or stable transition. Longitudinal single-cell and spatial approaches, paired with functional assays, may help determine whether observed states are transient, stable, or selected under stress.

Whether EV-associated signals causally and durably regulate EMP in human HGSOC remains unresolved. Studies should separate EV-mediated effects from soluble factors and determine whether a defined vesicle population and cargo are necessary and sufficient for the phenotype. Findings from preclinical models should be presented as mechanistic support, not evidence of clinical reversibility.

For EV-associated biomarkers, future studies should prioritize analytical reproducibility, independent clinical validation, comparison with CA-125 and imaging where appropriate, and evidence that a marker improves decision-making. Exploratory signatures and observational recurrence-monitoring studies should not be described as clinically validated tests without these steps.

The ascites environment warrants study as a heterogeneous mixture rather than a uniform EV niche. Patient-derived material varies in cellular sources, soluble mediators, particle composition, treatment exposure, and physical properties. Mechanistic experiments should characterize these factors and avoid assigning whole-ascites effects to EVs unless supported by fractionation, depletion/add-back, and cargo-specific evidence.

Finally, engineered EVs and EV-release/uptake inhibitors require evaluation for specificity, biodistribution, dose, safety, manufacturing consistency, and efficacy in clinically relevant models before clinical benefit can be inferred. Clinical trial identifiers, design, recruitment status, and stated objectives should be verified directly against authoritative registries before submission.

## 11. Conclusions

Overall, the literature supports a framework in which EV-associated communication may interact with tumour cell plasticity, ascites-associated stress, metastatic colonization, and treatment resistance in ovarian cancer. The evidence is not uniform: some studies provide functional evidence in cell or animal models, others report associations in patient samples, and many do not establish exosome-specific biogenesis or cargo-dependent causality. The conceptual contribution of this review is to connect these domains while explicitly distinguishing EVs from endosome-derived exosomes, EMT from reversible EMP states, and preclinical plausibility from clinical validation. This evidence-calibrated view identifies the experiments and clinical validation needed before proposed biomarkers or therapeutic strategies can be considered actionable.

## Data Availability

No new data were created or analyzed in this study. Data sharing is not applicable to this article.

## Abbreviations

The following abbreviations are used in this manuscript:

ABCB1	ATP-Binding Cassette Subfamily B Member 1
ADEs	Ascites-Derived Exosomes
AKT	Protein Kinase B
ALDH1	Aldehyde Dehydrogenase 1
AP-1	Activator Protein 1
CAFs	Cancer-Associated Fibroblasts
CA-125	Cancer Antigen 125
CBX7	Chromobox Protein Homolog 7
CCR1	C-C Chemokine Receptor Type 1
CDK8/19	Cyclin-Dependent Kinases 8 and 19
circRNAs	Circular RNAs
CSCs	Cancer Stem Cells
CTCs	Circulating Tumor Cells
CX3CR1	C-X3-C Motif Chemokine Receptor 1
DNMT1	DNA Methyltransferase 1
ECM	Extracellular Matrix
EMT	Epithelial–Mesenchymal Transition
EMP	Epithelial–Mesenchymal Plasticity
EpCAM	Epithelial Cell Adhesion Molecule
ESCRT	Endosomal Sorting Complex Required for Transport
EVs	Extracellular Vesicles
FAK	Focal Adhesion Kinase
FIGO	International Federation of Gynecology and Obstetrics
GPCRs	G Protein-Coupled Receptors
GROα	Growth-Regulated Oncogene Alpha
HE4	Human Epididymis Protein 4
HGSOC	High-Grade Serous Ovarian Carcinoma
HIF-1α	Hypoxia-Inducible Factor 1 Alpha
HPMCs	Human Peritoneal Mesothelial Cells
HSPGs	Heparan Sulfate Proteoglycans
HSP70	Heat Shock Protein 70
HSP90	Heat Shock Protein 90
IL-6	Interleukin-6
IL-8	Interleukin-8
ILVs	Intraluminal Vesicles
ILK	Integrin-Linked Kinase
lncRNAs	Long Non-Coding RNAs
MMPs	Matrix Metalloproteinases
MMT	Mesothelial-to-Mesenchymal Transition
mRNAs	Messenger RNAs
MVBs	Multivesicular Bodies
NF-κB	Nuclear Factor Kappa B
NK Cells	Natural Killer Cells
nSMase2	Neutral Sphingomyelinase 2
Oct-4	Octamer-Binding Transcription Factor 4
PDK4	Pyruvate Dehydrogenase Kinase 4
PI3K	Phosphoinositide 3-Kinase
PRRT2	Proline-Rich Transmembrane Protein 2
PSFs	Phenotypic Stability Factors
RAB13	Ras-Related Protein Rab-13
RAB25	Ras-Related Protein Rab-25
ROMA	Risk of Ovarian Malignancy Algorithm
siRNA	Small Interfering RNA
SMAD	Sma and Mad-Related Proteins
STAT3	Signal Transducer and Activator of Transcription 3
TAMs	Tumor-Associated Macrophages
TAZ	Transcriptional Coactivator with PDZ-Binding Motif
TGF-β	Transforming Growth Factor Beta
TGFβRI	Transforming Growth Factor Beta Receptor I
TGFβRII	Transforming Growth Factor Beta Receptor II
TME	Tumor Microenvironment
TP53	Tumor Protein p53
TWIST1	Twist Family BHLH Transcription Factor 1
VEGF	Vascular Endothelial Growth Factor
WGD	Whole-Genome Doubling
Wnt	Wingless/Integrated Signaling Pathway
YAP1	Yes-Associated Protein 1
ZEB1	Zinc Finger E-Box Binding Homeobox 1
ZEB2	Zinc Finger E-Box Binding Homeobox 2
ZO-1	Zonula Occludens-1
